# Exploring Bile-Acid Changes and Microflora Profiles in Chicken Fatty Liver Disease Model

**DOI:** 10.3390/ani14070992

**Published:** 2024-03-23

**Authors:** Wen-Yuan Yang, Pei-En Chang, Sin-Jin Li, Shih-Torng Ding, Yuan-Yu Lin

**Affiliations:** 1 Department of Animal Science and Technology, National Taiwan University, Taipei City 106, Taiwan; yangmei9427@gmail.com (W.-Y.Y.); sding@ntu.edu.tw (S.-T.D.); 2 Institute of Biotechnology, National Taiwan University, Taipei City 106, Taiwan; f09642004@ntu.edu.tw; 3 Bachelor Program of Biotechnology and Food Nutrition, National Taiwan University, Taipei City 106, Taiwan; sinjinli@ntu.edu.tw

**Keywords:** non-alcoholic fatty liver, bile acids, cecal microbiota, high-cholesterol low-choline diet

## Abstract

**Simple Summary:**

In this study, we investigated the impact of a high-cholesterol, low-choline diet on non-alcoholic fatty liver disease (NAFLD) in laying hens, a condition known to decrease egg production. Our research focused on bile-acid metabolism as a potential avenue for addressing NAFLD. Using 7-week-old ISA female chickens, we induced NAFLD through a 6-week diet intervention, analyzed serum and cecal bile acids through LC/MS, and conducted 16S rRNA sequencing on cecal digesta DNA. The results revealed significant differences in bile-acid distribution between healthy and diet-induced NAFLD chickens. Notably, the diet led to alterations in both serum and cecal bile-acid profiles, affecting synthesis enzymes in the liver. Microbiota analysis demonstrated distinct differences in abundance and composition between the two groups. This study sheds light on the changes in bile acid and microflora associated with a chicken NAFLD model, contributing valuable insights into fatty liver disease metabolism and offering potential targets for intervention.

**Abstract:**

Excessive liver fat causes non-alcoholic fatty liver disease (NAFLD) in laying hens, reducing egg production. Addressing NAFLD via bile-acid metabolism is gaining attention. We induced NAFLD in 7-week-old ISA female chickens with a high-cholesterol, low-choline diet (CLC) for 6 weeks. LC/MS was used to analyze serum and cecal bile acids, while cecal digesta DNA underwent 16S rRNA sequencing. The distribution of bile acid varied in healthy (CON) and CLC-fed chickens. CLC increased secondary bile acids (TLCA, TUDCA, THDCA, TDCA) in serum and primary bile acids (CDCA, TCDCA, isoDCA) in serum, as well as glycochenodeoxycholic acid (GCDCA) in cecal contents. CLC upregulated bile-acid synthesis enzymes (CYP7A1, CYP8B1) in the liver. Bile-acid receptor gene expression (HNF4A, FXR, LXR) was similar between groups. Microbiota abundance was richer in CON (alpha-diversity), with distinct separation (beta-diversity) between CON and CLC. The Firmicutes/Bacteroidetes ratio slightly decreased in CLC. Taxonomic analysis revealed higher *Bacteroides*, *Alistipes*, *Megamonas* in CLC but lower *Barnesiella*. CLC had more Mucispirillum, Eubacterium_coprostanoligenes_group, Shuttleworthia, and Olsenella, while CON had more Enterococcus, Ruminococcaceae_UCG_014, and Faecalibacterium. This study unveils bile-acid and microflora changes in a chicken NAFLD model, enhancing our understanding of fatty liver disease metabolism and aiding targeted interventions.

## 1. Introduction

Chylomicrons are called portomicrons in birds. Compared to mammals, birds have a less-lymphatic system. The hepatic portal carries the portomicrons to the liver for lipid metabolism, a characteristic that predisposes birds to deposit fat in the liver or even exposure to liver toxins [1,2,3]. Fatty liver disease is a common metabolic disease in poultry, and it is also one of the leading causes of non-infectious disease-related death in laying hens, and it is more likely to affect high-yielding hens [4]. The fatty liver symptoms caused by abnormal accumulation of lipids in poultry can be divided into fatty liver syndrome (FLS) and fatty liver hemorrhagic syndrome (FLHS). In high-producing hens, liver embrittlement ruptures and hemorrhages can increase mortality [5,6]. The aforementioned symptoms may be affected by feeding management, genetic background, or endocrines [7,8,9,10,11].

Bile acids provide the function of emulsifying lipids, cholesterol, and fat-soluble vitamins in the intestine. They also wrap these substances into chylomicrons, which facilitate the absorption of their nutrients by the intestinal epithelium. In addition, they also participate in glucose metabolism, lipid metabolism, and energy retention, and have endocrine functions that regulate their own biosynthesis. Bile acids are amphiphilic molecules with a structure similar to cholesterol, are synthesized by cholesterol in the liver, and are exerted by two different pathways: the classical and alternative pathways [12]. Two major enzymes—cytochrome P450 family 7 subfamily A member 1 (CYP7A1) and cytochrome P450 family 27 subfamily A member 1 (CYP27A1)—play regulatory roles in the two pathways. CA (cholic acid) and CDCA (chenodeoxycholic acid) are known as the primary bile acids, which means they are major products in these two pathways and can be modified by the gut microbiome [13] After modifying, the metabolites are called secondary bile acids, such as lithocholic acid (LCA) and deoxycholic acid (DCA) [13]. Some researchers found that total bile acids, including primary and secondary bile acids, elevate in serum from non-alcoholic fatty liver disease [14,15]. However, the composition of bile acid in chickens in the fatty liver model is unclear. The high-cholesterol and low-choline diet is an effective model of fatty liver disease in poultry [8]. In this study, we focus on exploring the changes in bile acid and the profile of microflora in the chicken fatty liver disease model.

## 2. Materials and Methods

### 2.1. Experimental Design

Animal experiments were approved by the Institutional Animal Care and Use Committee of National Taiwan University (NTU-108-EL-00024). A total of twenty 7-week-old female ISA chickens were obtained from commercial farms in Taiwan. Chickens were individually housed in battery cages. Each cage (45 cm long × 30 cm wide × 37 cm high) was utilized to house a single chicken. Water and feed were provided ad libitum. The lighting schedule was a 16 h light/8 h dark cycle throughout the experiment. The mean ambient temperature was 25 ± 3 °C; the relative humidity was maintained within the range of 60–70%. The chickens were allotted to two dietary treatments for 6 weeks: a basal diet (CON) and a high-cholesterol and low-choline diet (CLC). The diet ingredients and calculated nutritional values are shown in Table 1.

### 2.2. Sample Collection

At the end of the experiment, the cecal contents and blood samples were collected for further microbiome and bile-acid analysis. The hens were injected intravenously with sodium pentobarbital (30 mg/kg body weight), and cervical dislocation was performed. Approximately 1 g of homogeneous cecal contents was collected, aliquoted into two sterilized tubes, and stored at −80 °C. One of these cecal-content samples was used for DNA extraction and microbiota analysis, and the other samples were used for bile-acid analysis. Blood was collected from the left brachial vein of the hens; these blood samples were centrifuged at 1500× *g* for 15 min at 4 °C to collect serum. The serum sample was stored at −80 °C.

### 2.3. Bile-Acid Extraction and Analysis

Then, 100 μL serum or 200 mg cecal contents were extracted with 300 μL methanol containing an internal standard mixture, evenly mixed for more than 1 min, and stayed on ice for 30 min. Samples were centrifuged with 12,000× *g* for 30 min at 4 °C. The supernatant was transferred for bile-acid analysis. The analysis was performed using Waters ultra-high-performance liquid chromatography coupled with a Waters Xevo TQS MS (Waters Corp., Milford, MA, USA). For bile-acid analysis, chromatographic separation was performed on a Waters ACQUITY BEH C8 column (2.1 mm × 100 mm × 1.7 μm). The column temperature was maintained at 60 °C. For optimized parameters, mobile phase A was 10% acetonitrile with 0.01% formic acid, and mobile phase B was isopropanol/acetonitrile (50:50, *v*/*v*) with 0.01% formic acid. Mass analysis was performed using the Waters Xevo TQ-S system in positive-ion ESI mode. The capillary voltage was set at 1.5 KV. The desolvation gas flow rate was set at 1000 L/h, and the cone gas flow was maintained at 150 L/h. The desolvation and source temperatures were set at 600 °C and 150 °C, respectively. The QC sample (laboratory quality control) and mix QC sample (a mixture of all samples) were prepared for analysis during the analytical runs after every 10th sample.

### 2.4. RNA Extraction and Real-Time PCR Analysis

Total RNA was extracted from liver tissues using the TRIzol Reagent (Invitrogen, Carlsbad, CA, USA) in accordance with the manufacturer’s instructions. Samples were digested with DNase I (Ambion, Austin, TX, USA) at 37 °C for 30 min to remove genomic DNA interference and then reverse transcribed using a High-Capacity cDNA Reverse Transcription kit (Applied Biosystems, Foster City, CA, USA). The transcribed cDNA was amplified using a CFX96 Real-Time PCR Detection System (Bio-Rad, Richmond, CA, USA), and the end products were reacted with SYBR Green (Finnzymes, Espoo, Finland). Conditions for PCR reactions were initially denaturation at 95 °C for 7 min, followed by 39 cycles of denaturation at 95 °C for 10 s, and annealing at 60 °C for 30 s. The mRNA levels of each gene were normalized using PPIA levels in the same sample and calculated using the formula of 2^−(Ct target genes−Ct PPIA)^. The sequences of specific PCR primers for target gene amplification were as follows: peptidylprolyl isomerase A (*PPIA*), 5′-AGGTGCCCATAACAGCAGAG-3′ (forward) and 5′-CACCACCCTGACACATGAAG-3′ (reverse); cytochrome P450 family 7 subfamily A member 1 (*CYP7A1*), 5′-TAGCACCATGGATCTGGGGA-3′ (forward) and 5′-CCAAACTGCAAGGCACATCC-3′ (reverse); cytochrome P450 family 7 subfamily B member 1 (*CYP7B1*), 5′-ATGGCTGGGAGGGTCAAAAG-3′ (forward) and 5′-GCCCACAGGGCAAAATG-3′ (reverse); cytochrome P450 family 27 subfamily A member 1 (*CYP27A1*)*,* 5′-CGGAGACTAGGATCTGGGGA-3′ (forward) and 5′-ACGGACCCCATAGCCAAAAG-3′ (reverse); cytochrome P450 family 8 subfamily B member 1 (*CYP8B1*), 5′-ACGCACTGGACTTCAGACAG-3′ (forward) and 5′-ACGATGGCTCCAAAGCAGAA-3′ (reverse); hepatocyte nuclear factor 4 alpha (*HNF4A*), 5′-GAGCGTGAGGAAGAACCACA-3′ (forward) and 5′-TGCAGTATCGGCACTGGTTT-3′ (reverse); liver X receptor (*LXR*), 5′-GCAGCGTTTTGCTCACTTCA-3′ (forward) and 5′-CTGGATTGTAGCGCCGAGAT-3′ (reverse); farnesoid X receptor (*FXR*), 5′-GAGCGTGAGGAAGAACCACA-3′ (reverse) and 5′-TGCAGTATCGGCACTGGTTT-3′ (reverse).

### 2.5. DNA Extraction of and 16S rDNA Amplicon Pyrosequencing

The total bacterial genomic DNA in each cecal-content sample was extracted using the QIAamp Fast DNA Stool Mini Kit (QIAGEN, Hilden, Germany). The extracted DNA was then measured using a SimpliNano spectrophotometer (Biochrom, Cambridge, UK) and agarose gel electrophoresis. Then, paired-end 2 × 300 bp sequencing was performed using the Illumina MiSeq platform with a MiSeq Reagent Kit (Illumina, San Diego, CA, USA).

### 2.6. Sequence Analysis

De-multiplexing was carried out based on barcode identification. As a quality control, reads with a Q score less than the threshold (Q < 20) were discarded in the QIIME 1.9.1 pipeline (Caporaso et al., 2010 [16]). If three consecutive bases were <Q20, the read was truncated, and the resulting read was retained in the data set only if it was at least 75% of the original length using split_libraries_fastq.py script in QIIME [17]. Sequences were chimera-checked using UCHIME to obtain the effective tags [18] and filtered from the data set before operational taxonomic unit (OTU) clustering at 97% sequence identity using the UPARSE [19] function in the USEARCH v.7 pipeline [20]. For each representative sequence, the RDP classifier (v.2.2) algorithm [21] was employed to annotate taxonomy classification based on the information retrieved from the Silva Database v.132 [22,23].

To normalize the variations in sequence depth across samples, OTU abundance information was rarefied to the minimum sequence depth using the QIIME script (single_rarefaction.py). Subsequent analysis of both alpha- and beta-diversities was performed using the normalized data. Alpha diversity was indicative of the species complexity within individual samples based on different criteria output from the QIIME pipeline. Community richness was also assessed by the Chao1 and ACE indices.

Beta-diversity analysis was used to evaluate the differences among samples in terms of species complexity. A cluster analysis was preceded by a principal component analysis (PCA), which was applied to reduce the dimensions of the multiple variables using the FactoMineR package and ggplot2 package in R software (v.2.15.3). To further increase the group distinction, the supervised partial-least-squares discriminant analysis (PLS-DA) was used to evaluate and visualize variance based on the OTU level of gut microbiota composition among the groups. PLS-DA was performed using the R package mixOmics.

Statistically significant biomarkers were identified by the use of the LEfSe analysis [24]. LEfSe applies LDA to those bacterial taxa identified as significantly difference and further assesses the effect size of each differentially abundant taxon. In this study, taxa with an LDA score (log 10) > 3 were considered significant.

### 2.7. Statistical Analysis

The Kolmogorov–Smirnov test was used to test the normal distribution of the data before statistical analysis was performed. Statistical analyses were performed using GraphPad software (version 5 for Windows). The collected data were tested by means of an unpaired Student’s *t*-test. Significance was declared at *p* ≤ 0.05.

## 3. Results

### 3.1. Bile-Acid Profile in Serum and Cecal Contents

Bile-acid levels in the serum and cecal contents of healthy control (CON) chickens and chickens with non-alcoholic fatty liver disease fed a high-cholesterol, low-choline (CLC) diet were measured. The comparison of bile-acid profiles between CON and CLC chickens is summarized in Table 2 and Table 3. Significant differences were observed in the distribution of bile-acid pools between the CON and CLC groups. Total concentrations of secondary bile acids were increased in the CLC group, including taurolithocholic acid (TLCA), tauroursodeoxycholic acid (TUDCA), taurohyodeoxycholic acid (THDCA), and taurodeoxycholic acid (TDCA). Regarding primary bile acids, chenodeoxycholic acid (CDCA), taurochenodeoxycholic acid (TCDCA), and 3β-hydroxydeoxycholic acid (isoDCA) showed significantly higher levels in the serum of the CLC group compared to the CON group. In the cecal contents, the CLC group exhibited significantly higher levels of glycochenodeoxycholic acid (GCDCA), a primary bile acid, compared to the CON group. The serum profile revealed that TCDCA took the lead in the bile-acid pool (Table 2), while TCA showed the greatest proportion in cecal content (Table 3). In particular, the concentration of TLCA was elevated approximately 75-fold in the CLC group and 80-fold in CLC cecal contents. Interestingly, most of the secondary bile-acid species exhibited significant increases both in serum and cecal contents of CLC.

### 3.2. Hepatic Gene Expression

**Figure 1:**
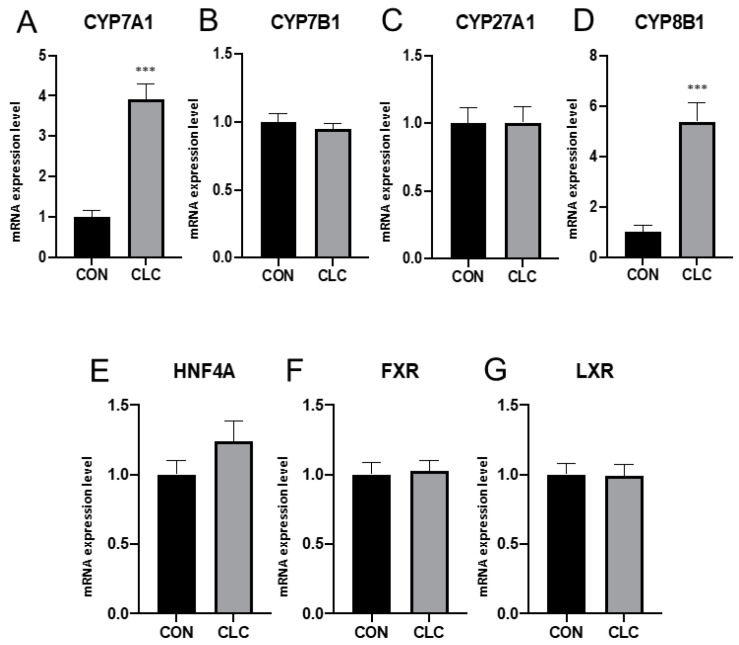
Expression of bile-acid metabolic genes in liver tissue. (**A**) CYP7A1, cytochrome P450 family 7 subfamily A member 1 (**B**) CYP7B1, cytochrome P450 family 7 subfamily B member 1 (**C**) CYP27A1, cytochrome P450 family 27 subfamily A member 1 (**D**) CYP8B1, cytochrome P450 family 8 subfamily B member 1 (**E**) HNF4A, hepatocyte nuclear factor 4 alpha (**F**) FXR, farnesoid X receptor (**G**) LXR, liver X receptor. Unpaired *t*-test, symbols indicate statistical significance, *** *p* ≤ 0.001. CON: control; CLC: high cholesterol with low choline. Data presented as mean ± S.D. (*n* = 10).

To explore the hepatic gene expression related to bile-acid metabolism, here, we examined the mRNA level from CON and CLC chickens. The expression of CYP7A1, the major enzyme response for bile-acid synthesis, showed a four-fold increase in CLC livers (Figure 1A). Upregulated expression was also found in CYP8B1 (Figure 1B), while there was no change in CYP7B1 and CYP27A1 (Figure 1C,D). Further, we examined the expression level of bile-acid receptor genes. However, no significant difference was observed in HNF4A, FXR, and LXR between CON and CLC (Figure 1E–G).

### 3.3. Effect of CLC Diet on Cecal Digesta Microbiota

**Figure 2:**
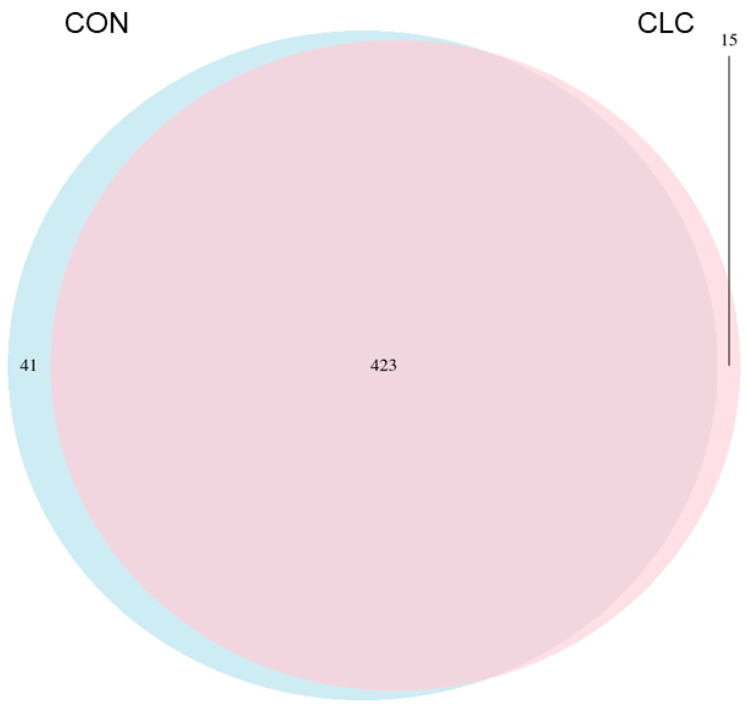
The Venn diagram analysis of 479 OTUs in the cecum of hens. A total of 41 and 15 OTUs were found in control and CLC groups, respectively. Furthermore, 423 OTUs were found in the control and CLC groups. CON: control group with basal diet; CLC: CLC group with high-cholesterol and low-choline diet. (*n* = 10).

**Figure 3:**
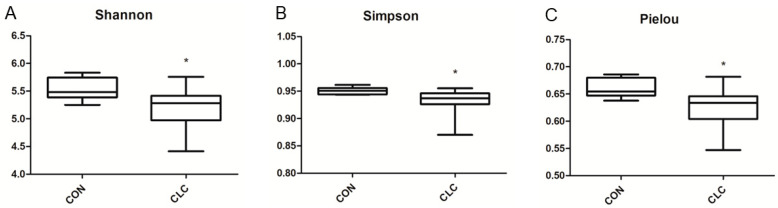
Differences in bacterial community diversity in the cecum of hens between CON and CLC group. The diversity indices of (**A**) Shannon, (**B**) Simpson and (**C**) Pielou are shown. CON: control group with basal diet; CLC: CLC group with high-cholesterol and low-choline diet. * *p* ≤ 0.05. Data presented as mean ± S.D. (*n* = 10).

**Figure 4:**
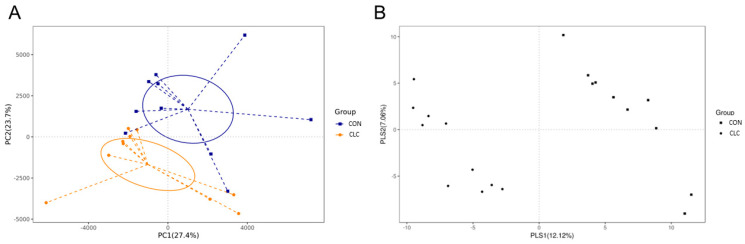
The similarity of the identified OTUs between groups: (**A**) Principal components analysis (PCA) of the bacterial community structure between control and CLC group. Each symbol represents each gut microbiota; (**B**) partial-least-squares discriminant analysis (PLS-DA). Each symbol represents each gut microbiota. Square symbol represents control group; circle symbol represents CLC group. CON: control; CLC: high cholesterol with low choline. (*n* = 10).

**Figure 5:**
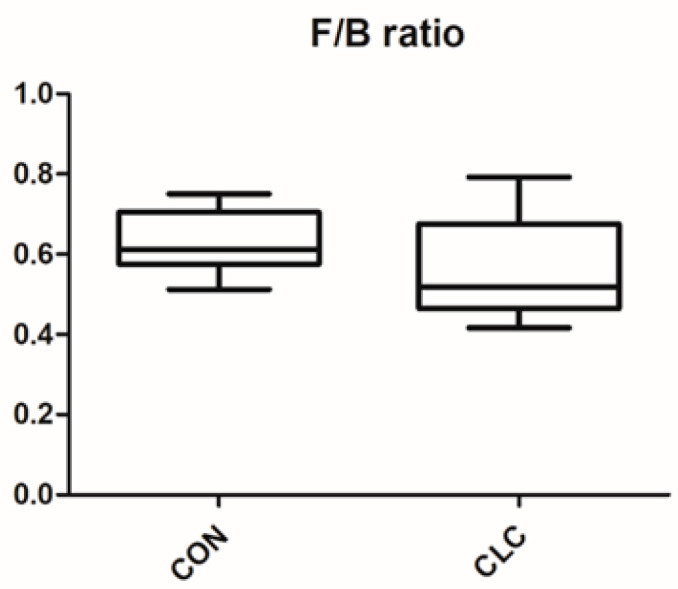
Effect of high-cholesterol and low-choline diet on gut Firmicutes/Bacteroidetes (F/B) ratios. CON: control; CLC: high cholesterol with low choline. Data presented as mean ± S.D. (*n* = 10).

Here, we determined the gut microbiome composition to clarify the influence between the intestine and the liver. A total of 479 OTUs were found in cecal contents, shown in a Venn diagram in Figure 2. Among these OTUs, 423 were commonly observed in both groups, whereas 41 OTUs existed only in CON and 15 OTUs existed only in CLC. For alpha-diversity, the abundance of microbiota was significantly enriched in CON (*p* < 0.05), which was assessed by the Shannon, Simpson, and Pielou indices (Figure 3). For beta-diversity, a principal component analysis (PCA) based on weighted Unifrac demonstrated that OTUs from CON and CLC were distinctly separated (Figure 4A, PC1:27.4%; PC2:23.7%). Similar to PCA, a partial-least-squares discriminant analysis (PLS-DA) illustrated the structural differences between groups. (Figure 4B, PLS1:12.12%; PLS2:7.06%). Compared to CON, CLC tended to have a lower Firmicutes/Bacteriodetes (F/B) ratio; nevertheless, it showed no significance (Figure 5).

### 3.4. Effect of CLC Diet on Bacterial Taxonomic Composition of Cecal Digesta

**Figure 6:**
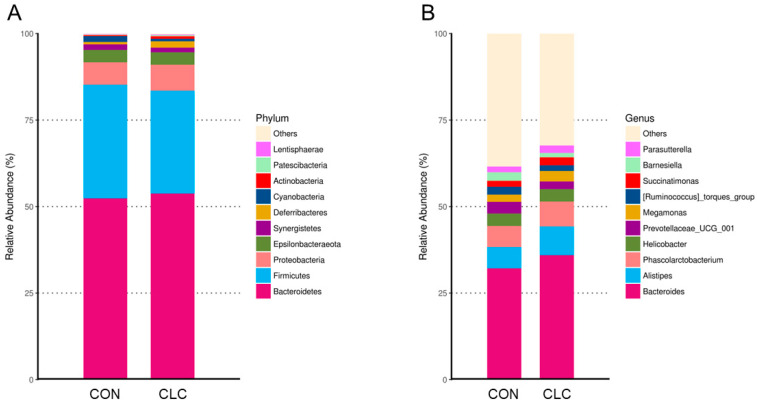
The relatively abundant microbiota in different taxonomic levels. (**A**) Phylum level. (Bacteroidetes—CON: 52.38%, CLC: 53.76%; Firmicutes—CON: 38.26%, CLC: 29.73%; Proteobacteria—CON: 6.47%, CLC: 7.49%; Epsilonbacteraeota—CON: 3.60%, CLC: 3.64%; Synergistetes—CON: 1.57%, CLC: 1.28%; Deferribacteres—CON: 0.73%, CLC: 1.89%; Cyanobacteria—CON: 1.67%, CLC: 0.76%; Actinobacteria—CON: 0.26%, CLC: 0.68%; Patescibacteria—CON: 0.20%, CLC: 0.31%; Lentisphaerae—CON: 0.14%, CLC: 0.21%; Others—CON: 0.12%, CLC: 0.24%) (**B**) Genus level. (Bacteroides—CON: 32.13%, CLC: 35.96%; Alistipes—CON: 6.16%, CLC: 8.28%; Phascolarctobacterium—CON: 6.11%, CLC: 7.19%; Helicobacter—CON: 3.60%, CLC: 3.64%; Prevotellaceae_UCG_001—CON: 3.37%, CLC: 2.20%; Megamonas—CON: 2.06%, CLC: 3.07%; [Ruminococcus]_torques_group—CON: 2.30%, CLC: 1.65%; Succinatimonas—CON: 1.70%, CLC: 2.23%; Barnesiella—CON: 2.50%, CLC: 1.32%; Parasutterella—CON: 1.62%, CLC: 2.13%; Others—CON: 38.46%, CLC: 32.35%. Each color represents a different taxonomic unit (*n* = 10). CON: control; CLC: high cholesterol with low choline.

The bacterial taxonomy composition in the cecal contents of chickens was analyzed, and the results are depicted in Figure 6. The analysis revealed that the relative abundance of the phyla Firmicutes was 32.9% in the control group (CON) and 29.7% in the experimental group (CLC). Similarly, the phylum Bacteroidetes accounted for 52.4% in the CON group and 53.8% in the CLC group. The relative abundance of the phyla Proteobacteria was 6.5% in the CON group and 7.4% in the CLC group, while the phylum Epsilonbacteraeota comprised 3.6% in both the CON and CLC groups (Figure 6A). At the genus level, we observed an elevated abundance of the genera *Bacteroides*, *Alistipes*, and *Megamonas* in the CLC group compared to the CON group. However, a contrasting trend was observed for the genus *Barnesiella* (Figure 6B). In addition, the microbiota analysis showed that Firmicutes and Bacteroidetes are the most common phyla in chicken ceca, and Proteobacteria, Epsilonbacteraeota, and Synergistetes account for the remainder (Figure 6A).

**Figure 7:**
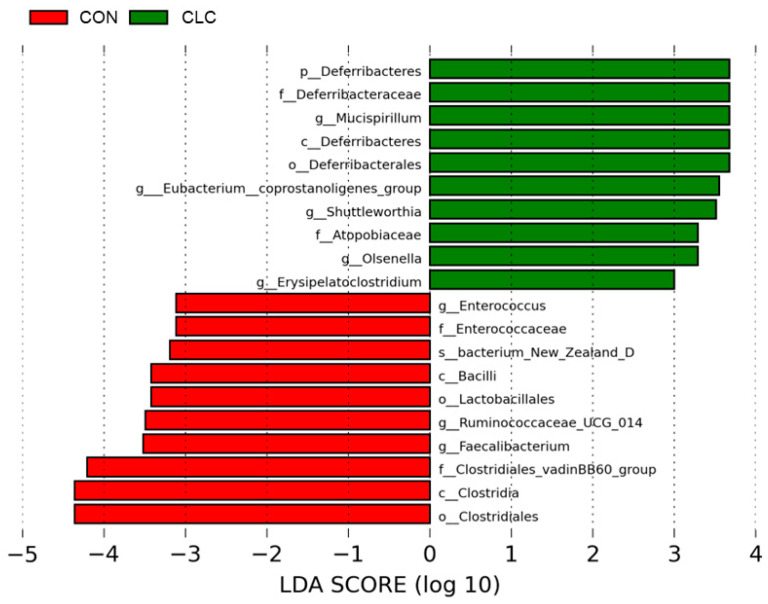
LEfSe analysis showing the most differentially abundant taxa between control and CLC group. Only taxa with LDA > 3 are shown. The letter in front of the strains indicates the taxon level; p, phylum; c, class; o, order; f, family; g, genus; s, species (*n* = 10). CON: control; CLC: high cholesterol with low choline.

**Figure 8:**
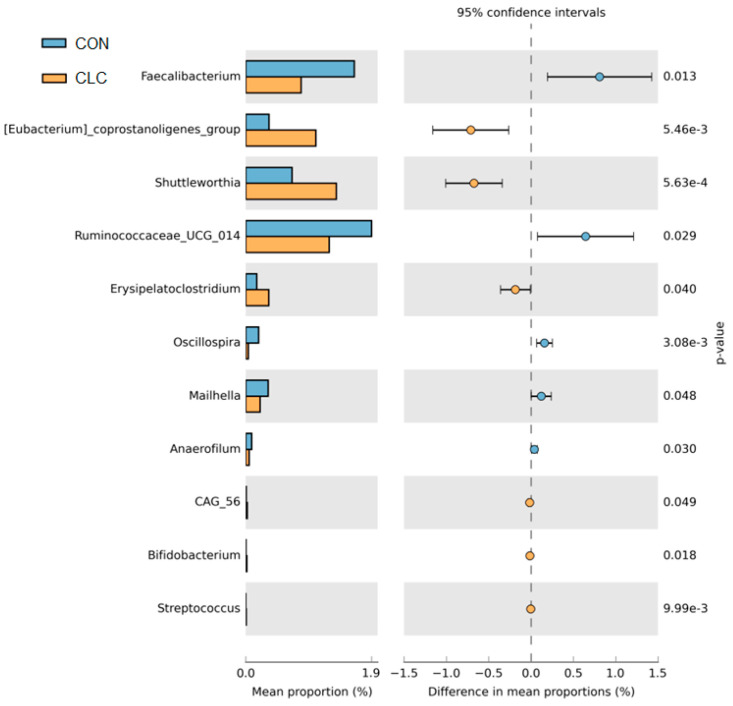
The Welch’s test of group CON and CLC. CON: control; CLC: high cholesterol with low choline. Data presented as mean ± S.D. (*n* = 10).

Figure 7 presents the bacterial taxa that exhibited significant differences (*p* ≤ 0.05) between the CON and CLC groups, as determined by LEfSe and LDA analysis. The CLC group displayed a higher abundance of *Mucispirillum*, *Eubacterium_coprostanoligenes_group*, *Shuttleworthia*, and *Olsenella*, while the CON group exhibited a higher abundance of *Enterococcus*, *Ruminococcaceae_UCG_014*, and *Faecalibacterium*. Additionally, we performed Welch’s *t*-test to compare the abundance of gut bacteria between the two groups and identify those with significant differences (*p* ≤ 0.05), as illustrated in Figure 8. The CON group demonstrated a significantly higher abundance of *Faecalibacterium*, *Ruminococcaceae_UCG_014*, *Oscillospira*, *Maihella,* and *Anaerofilum*. Conversely, the CLC group displayed a significantly higher abundance of *[Eubacterium]_ coprostanoligenes_group*, *Shuttleworthia*, and *Erysipelatoclostridium*.

## 4. Discussion

This study provides the first elucidation of changes in bile acids in non-alcoholic fatty liver in laying hens and reveals that fatty liver induced by CLC feed leads to an increase in secondary bile acids. Furthermore, bile-acid analysis data show that chickens tend to conjugate bile acids with taurine rather than glycine. Conjugated bile acids, such as glycine and taurine, exhibit lower pK_a_ dissociation constants, which reduces the hydrophobicity of bile acids, preventing their accumulation in the liver and subsequent toxicity. Conjugation with glycine or taurine also enhances bile-acid stability and protects against pancreatic enzyme hydrolysis [25]. Many studies have shown species-specific preferences for conjugated amino acids. For instance, in humans, rabbits, and guinea pigs, bile-acid conjugation is primarily with glycine, while in mice, sheep, and dogs, it is predominantly with taurine [26,27]. Our experimental results indicate that in laying hens, bile acids tend to conjugate with taurine rather than glycine. The reasons for these species-specific preferences for different amino acids remain unclear. Since chickens primarily utilize taurine as the main conjugating amino acid for bile acids, it is worth noting that taurine possesses various physiological functions, such as preventing oxidative stress-induced damage, lipid degeneration, inflammation, and alcohol-induced injury. The conjugation and deconjugation of bile acids are related to intestinal microbiota. Bile salt hydrolase, an enzyme responsible for bile-acid deconjugation, is expressed abundantly by certain anaerobic bacterial groups, including *Bacteroides*, *Lactobacillus*, *Clostridium*, and *Bifidobacterium* [13]. However, whether bacteria that preferentially utilize taurine are included among these groups remains unclear.

Previous studies have indicated differences in gut microbiota composition between patients with non-alcoholic fatty liver disease and healthy individuals. For example, Jiao et al. [13] demonstrated higher levels of bacteria such as *Escherichia*, *Bilophila*, and *Rhodobacter* in the intestines of non-alcoholic fatty liver patients compared to the control group. In our study, we observed changes in gut microbiota in the CLC group, which was fed a high-cholesterol, low-choline diet, compared to the control group. Specifically, the relative abundance of the *Bacteroides* genus was higher in the CLC group. *Bacteroides* are the predominant anaerobic bacteria in the gut, exhibiting bile-acid tolerance and the ability to convert carbohydrates into volatile fatty acids, serving as an energy source for the host [28]. *Bacteroides* also contribute to the development of lymphoid tissues in the gut, enhancing immune function [29], but an overabundance of *Bacteroides* may lead to intra-abdominal infections and intestinal inflammation [30]. In addition to the *Bacteroides* genus, the *Alistipes* genus was also found to be more abundant in the CLC group compared to the control group. *Alistipes* have been shown to exhibit resistance to bile acids and can survive in high concentrations of bile acids [31,32]. Some studies have suggested that changes in *Alistipes* abundance and gut dysbiosis are associated with non-alcoholic fatty liver disease and liver fibrosis [33,34]. Furthermore, a specific subspecies of *Alistipes*, *Alistipes finegoldii*, has been demonstrated to promote intestinal health in broiler chickens and act as a growth promoter [35]. *Alistipes* has been shown to possess anti-inflammatory properties, and its isomerase enzymes act on acetyl-CoA carboxylase, converting acetyl-CoA to malonyl-CoA, thereby generating propionic acid and acetic acid in the cecal microbiota of chickens. These metabolites are involved in the citric acid cycle and lipid metabolism, producing short-chain fatty acids that exhibit anti-inflammatory effects [36]. Therefore, the high-fat diet associated with the CLC feeding regimen may contribute to the relatively higher abundance of *Alistipes* in the CLC group. In the LEfSe analysis, we observed that the CLC group exhibited a higher abundance of *Mucispirillum*. Similar findings have been reported in rats fed a high-fat diet [37]. Additionally, in the Welch’s test, we identified higher levels of *Shuttleworthia* and *Erysipelatoclostridium* in the CLC group compared to the control group. Notably, *Erysipelatoclostridium* includes a species known for its high pathogenicity, causing various clinical conditions such as bacteraemia, septicaemia, brain and lung abscesses, and other infections affecting different organs and tissues. This species was previously referred to as *Bacillus ramosum* and *Clostridium ramosum*, and it was renamed *Erysipelatoclostridium ramosum* [38,39].

In our study, we observed a significant upregulation of hepatic genes CYP7A1 and CYP8B1 in the chickens fed the high-cholesterol, low-choline diet, indicating altered bile-acid metabolism in response to the dietary intervention. These findings raise important questions regarding the underlying mechanisms driving these gene expression changes and their implications for NAFLD pathogenesis. While the canonical bile-acid receptors FXR and TGR5 are well-established regulators of bile-acid metabolism, it is important to note that several other receptors are also involved in bile-acid signaling pathways. These receptors, including the Vitamin D Receptor, Pregnane X Receptor, Liver X Receptor α/β, Constitutive Androstane Receptor, Peroxisome Proliferator-Activated Receptors, and Retinoid X Receptor, have been shown to modulate bile-acid metabolism or be activated by bile acids themselves [40]. Our gene expression analysis suggests that the observed upregulation of CYP7A1 and CYP8B1 may not solely be mediated by the FXR pathway.

To date, our understanding of the variations in bile-acid profiles and alterations in the gut microbiota in chicken fatty liver disease models has been limited. However, preliminary results from this study pave the way to further investigations into the underlying mechanisms. Overall, our findings clarify the modifications to bile-acid profiles and the composition of the microflora in this model, enhancing our understanding of the metabolic disturbances linked to fatty liver disease. These insights might impact the development of targeted interventions aimed at mitigating the progression of this disease.

## 5. Conclusions

In summary, our study delves into the impact of a high-cholesterol, low-choline diet on NAFLD in laying hens, offering novel insights into potential interventions. Notably, our findings reveal distinct changes in bile-acid profiles, with an upregulation of secondary bile acids in serum and primary bile acids in both serum and cecal contents in response to the diet. Moreover, hepatic gene expression analysis unveiled notable increases in bile-acid synthesis enzymes, underscoring the metabolic shifts induced by the diet intervention. Microbiota analysis further elucidated differences in microbial abundance and composition between healthy and NAFLD chickens, highlighting potential links between gut dysbiosis and liver pathology. These findings contribute to a deeper understanding of NAFLD pathogenesis in chickens, with implications for both avian health and human nutrition strategies. Future studies may capitalize on these insights to develop targeted interventions aimed at mitigating fatty liver disease in poultry and beyond.

## Figures and Tables

**Table 1:** Diet composition and calculated nutrition levels.

Ingredient, %	Control	CLC
Corn Meal	67.08	67.08
Soybean Protein (36%)	8.05	8.05
Soybean Meal (44%)	9.84	9.84
Wheat Bran	4.47	4.47
Rice Bran	4.415	2.465
CaCO_3_	2.24	2.24
CaHPO_4_	1.79	1.79
Beef Tallow	1.34	1.34
NaCl	0.36	0.36
DL-Methionine	0.36	0.36
Premix ^1^	0.005	0.005
Choline	0.05	-
Cholesterol	-	2.00
Total	100	100
Nutrient Composition, %		
Crude Protein	16.55	16.94
Crude Fat	4.72	4.72
Methionine	0.63	0.63
Methionine + Cystine	0.91	0.91
Choline, mg/kg	0.13	0.08
ME, kcal/kg ^2^	3043	3043

^1^ Supplied per kg of diet: Vit. A, 1.8 mg; Vit. D3, 0.005 mg; Vit. E, 9.09 mg; Vit. K, 0.5 mg; Vit. B12, 0.007 μg; pantothenic acid, 2.99 mg; riboflavin, 1.63 mg; Cu, 1.25 mg; Mn, 24.06 mg; Zn, 12.7 mg; Se, 0.06 mg; Iodide, 0.35 mg. ^2^ Calculated compositions of crude protein, crude fat, methionine, cystine, choline and ME in diets.

**Table 2:** Bile-acid profile of serum in control and CLC group.

	CON (*n* = 10)	CLC (*n* = 10)
Primary Bile Acids (nM)		
TCA	122.3 ± 34.64	82.07 ± 17.26
CDCA	3.93 ± 1.52	38.13 ± 13.21 *
GCA	0.58 ± 0.11	0.47 ± 0.08
TCDCA	6080 ± 1153	14,556 ± 1868 **
isoDCA	0.25 ± 0.16	3.21 ± 0.59 **
T-α-MCA	9.39 ± 2.21	16.23 ± 3.34
Secondary Bile Acids (nM)		
TLCA	28.43 ± 7.77	2123 ± 481.8 ***
TUDCA	0.42 ± 0.10	13.37 ± 3.91 *
THDCA	19.35 ± 3.21	63.66 ± 6.99 ***
TDCA	1.51 ± 0.24	35.06 ± 7.67 **
5β-Cholenic Acid-7α-ol-3-one	1.00 ± 0.07	3.87 ± 0.41 ***

Unpaired *t*-test, symbols indicate statistical significance, * *p* ≦ 0.05, ** *p* ≦ 0.01, *** *p* ≦ 0.001. Data are represented as mean ± SEM. TCA, taurocholic acid; CDCA, chenodeoxycholic acid; GCA, lycocholic acid; TCDCA, taurochenodeoxycholic acid; isoDCA, 3β-hydroxydeoxycholic acid; T-α-MCA, tauro alpha-Muricholic acid sodium salt; TLCA, taurolithocholic acid; TUDCA, tauroursodeoxycholic acid; THDCA, taurohyodeoxycholic acid; TDCA, taurodeoxycholic acid.

**Table 3:** Bile-acid profile of cecal contents in control and CLC group.

	CON (*n* = 10)	CLC (*n* = 10)
Primary Bile acid (nM)		
CA	315.4 ± 133	116.7 ± 38.26
CDCA	1289 ± 369	1151 ± 293
GCDCA	8.25 ± 2.26	21.35 ± 4.04 *
GCA	1.72 ± 0.60	0.711 ± 0.10
TCA	10,300 ± 3118	12,302 ± 2208
T-α-MCA	447 ± 196.4	785 ± 169.6
HCA	1.197 ± 0.59	0.37 ± 0.10
Secondary Bile acid (nM)		
HDCA	0.56 ± 0.23	1.717 ± 0.34 *
UDCA	9.21 ± 2.57	11.88 ±1.96
LCA	4.32 ± 0.82	22.88 ± 6.85 **
isoLCA	3.48 ± 2.58	3.62 ± 1.51
TLCA	109.8 ± 27.36	8897 ± 1701 ***
TUDCA	15.82 ± 6.19	985 ± 377 *
THDCA	718.7 ± 219.2	3084 ± 502.9 ***
TDCA	10.12 ± 2.65	172.3 ± 37.41 ***
THCA	285.2 ± 76.32	131.8 ± 37.3
5β-Cholenic Acid-7α-ol-3-one	13.47 ± 8.16	8.07 ± 3.64
6,7-diketoLCA	1.54 ± 0.26	3.139 ± 0.40 *
7-ketoLCA	15.01 ± 6.69	11.81 ± 4.39

Unpaired *t*-test, symbols indicate statistical significance, * *p* ≦ 0.05, ** *p* ≦ 0.01, *** *p* ≦ 0.001. Data are represented as mean ± SEM. CA, cholic acid; CDCA, chenodeoxycholic acid; GCDCA, Glycochenodeoxycholic acid; GCA, lycocholic acid; TCA, taurocholic acid; T-α-MCA, tauro alpha-Muricholic acid sodium salt; HCA, hyocholic acid; HDCA, Hyodeoxycholic acid; UDCA, ursodeoxycholic acid; LCA, lithocholic acid; isoLCA, isolithocholic acid; TLCA, taurolithocholic acid; TUDCA, tauroursodeoxycholic acid; THDCA, taurohyodeoxycholic acid; TDCA, taurodeoxycholic acid; THCA, taurohyocholic acid sodium salt.

## Data Availability

The data that support the findings of this study are available from the corresponding author upon reasonable request. The raw data of reads presented in this study can be found in online repositories. The names of the repository/repositories and accession number(s) can be found below: Zenodo, target URL: https://doi.org/10.5281/zenodo.8332047 (accessed on 10 September 2023).
